# Effectiveness of the Stroke e-Learning Module on Malaysian Doctors’ Knowledge of Acute Ischaemic Stroke Management

**DOI:** 10.21315/mjms2024.31.4.16

**Published:** 2024-08-27

**Authors:** Stephenie Ann Albart, Abdul Hanif Khan Yusof Khan, Aneesa Abdul Rashid, Wan Asyraf Wan Zaidi, Irene Looi, Fan Kee Hoo

**Affiliations:** 1 Clinical Research Centre, Hospital Seberang Jaya, Seberang Jaya, Pulau Pinang, Malaysia; 2 Department of Neurology, Faculty of Medicine and Health Sciences, Universiti Putra Malaysia, Selangor, Malaysia; 3 Department of Family Medicine, Faculty of Medicine and Health Sciences, Universiti Putra Malaysia, Selangor, Malaysia; 4 Department of Medicine, Hospital Canselor Tuanku Muhriz, Universiti Kebangsaan Malaysia, Kuala Lumpur, Malaysia; 5 Department of Medicine, Hospital Seberang Jaya, Pulau Pinang, Malaysia

**Keywords:** stroke, acute stroke management, knowledge, Stroke e-Learning Module, doctors, questionnaire

## Abstract

**Background:**

The Stroke e-Learning Module (SEM) is a nationwide initiative to improve stroke care in Malaysia. This study aimed to assess the module’s effectiveness in improving knowledge on acute ischaemic stroke (AIS) management among Malaysian doctors.

**Methods:**

This was a pre-post study design. Medical officers, specialists and general practitioners from various disciplines who work in healthcare facilities in Malaysia were recruited virtually from those who registered for the SEM on the Docquity platform between 1 February 2021 and 31 January 2022. The Acute Stroke Management Questionnaire (ASMaQ), an existing validated questionnaire, was used to measure the doctors’ knowledge of AIS management before and after the SEM. The ASMaQ had three domains: i) general stroke knowledge (GSK), ii) hyperacute stroke management (HSM) and iii) advanced stroke management (ASM). The paired *t-* and the McNemar-tests were used to evaluate the effectiveness of the module.

**Results:**

One hundred and seventy-one participants voluntarily responded to the pre- and post-module questionnaires. The paired *t-*test revealed statistically significant improvement for the ASM knowledge scores (mean difference = 2.5; 95% CI: 1.8, 3.2; *P* < 0.001). The baseline proportion of participants with good knowledge of GSK, HSM and ASM were 92.4%, 64.9%, and 76%, respectively. The McNemar test showed that approximately 14% of the participants had significant improvement in ASM knowledge (*P* < 0.001). However, no significant changes were noted for GSK (−0.6%) and HSM (4.1%).

**Conclusion:**

The SEM has been shown to increase Malaysian doctors’ knowledge on ASM. However, greater effort should be made to improve GSK and HSM knowledge, particularly in areas related to stroke thrombolysis.

## Introduction

Stroke is the second major cause of disability and the third leading cause of mortality in Malaysia, with the incidence of ischaemic stroke increasing by 29.5% annually and the mortality rate is estimated at 7.6 per 100,000 (1, 2).

Disability-related stroke is potentially reversible if an eligible stroke patient receives reperfusion therapy within the recommended therapeutic window. Despite the availability of reperfusion therapy for acute ischaemic stroke (AIS) management in Malaysia, many patients failed to receive it within the stipulated time (3). Time is critical when managing AIS, as delayed treatment may lead to permanent disability and even death.

A previous study has shown that the most common reasons for prehospital delay in AIS management are the lack of awareness of stroke symptoms and failure to involve the emergency medical services (4). In-hospital delay factors such as physician’s reluctance, long interdepartmental communication times, lack of dedicated medical personnel and a lack of urgency in the management of AIS in the emergency department, were identified as barriers to initiating reperfusion therapy for stroke patients (5). These challenges are probably due to the lack of knowledge and training among healthcare professionals (HCPs) on the management of AIS.

AIS management in Malaysia has been rapidly evolving. A new clinical practice guideline (CPG) was published in 2021 to educate HCPs on the latest management of AIS (6). According to a study, approximately one-fourth of Malaysian HCPs had inadequate knowledge of AIS management, and stroke education needs to be incorporated for the subsequent guidelines (7).

Public education about stroke is also necessary, but it will not be sufficient to prevent treatment delays. HCP education is equally crucial. Every HCP plays an important role in educating stroke patients, identifying stroke symptoms and referring them early for thrombolysis therapy. They should be equipped with adequate knowledge and undergo the necessary training to be more aware of the current AIS management.

Several stroke education programmes have been shown to be effective in improving HCPs’ knowledge on acute ischaemic stroke management in various countries (8–11). The use of e-learning platform in the healthcare system has risen dramatically, especially after the COVID-19 pandemic. Even in times of crisis, continuing education and training among HCPs remain essential. An online stroke education programme, known as the Stroke e-Learning Module (SEM), was introduced in Malaysia in 2021 with the main goal of improving the quality of stroke care nationwide (12). The module was developed in collaboration with the Angels Initiative, based on the current Malaysian CPG on the management of AIS. It was aimed at raising the HCPs’ awareness and knowledge of AIS management; however so far, no studies have been done to show the effectiveness of the SEM.

Therefore, we aimed to determine the effectiveness of the SEM by assessing the HCPs’ knowledge of AIS management before and after the modules.

## Methods

### Study Design and Study Participants

This study used a pre-post study design. Participants’ knowledge was measured using an online questionnaire before and after the SEM (study intervention) was delivered. The eligible study participants were medical officers, specialists and general practitioners from various disciplines who work in healthcare facilities in Malaysia, regardless of private or government sectors. The study participants were recruited virtually among those who registered for SEM on the Docquity platform between 1 February 2021 and 31 January 2022. This is a follow-up study of the same participants of a previous study (7). Participants in the study provided written informed consent for answering both the pre- and post-module questionnaire. We emailed all the participants to complete the SEM and to answer the post-SEM questionnaire. The post-SEM questionnaire measures the post-module knowledge scores after the SEM was delivered using the ASMaQ. Reminder emails were sent every 2 weeks to remind the participants to complete the modules and to answer the post-SEM questionnaire.

### Study Intervention (Stroke e-Learning Module)

The SEM is the first self-learning online module on acute stroke management in Malaysia, developed by the Malaysia Stroke Council (MSC). This online module aims to increase awareness and knowledge about acute stroke management among Malaysian doctors. The modules were developed based on the Malaysian Clinical Practice Guideline (CPG) on the Management of Ischemic Stroke 2020 (3rd edition) that was published by the Malaysian Society of Neuroscience (MSN) in 2021. This module was made accessible to all HCPs via the Docquity platform (app.docquity.com) during the study period and is currently available on the Malaysia Stroke Council’s webpage (12). The module can be viewed at any time and can be paused, resumed or repeated as needed.

The online module comprised 10 topics covering various aspects of stroke management, such as pre-hospital care, hyperacute stroke management (HSM), post-stroke management and imaging. The module was delivered via pre-recorded video lectures by experts in the fields of neurology, internal medicine, family medicine and radiology. The average length of each module was approximately 30 min–40 min. The list of speakers and their credentials is shown in detail at the end of this manuscript (Appendix).

### Data Measurement

The pre-module questionnaire link consisted of two sections. Section 1 collected data on demographic variables such as age, gender, years of service, profession, work setting (primary care versus hospital), work sector (private versus government), treating stroke patients in daily practice and working with specialists. Section 2 measured the pre-module knowledge scores. This study used the Acute Stroke Management Questionnaire (ASMaQ), an existing validated questionnaire, to measure the knowledge on AIS management among HCPs (13). Permission to use this questionnaire was obtained from the copyright holders. It consisted of 29 items and had three domains: i) general stroke knowledge (GSK), ii) HSM and iii) advanced stroke management (ASM). The content validity was done by an expert panel and construct validity was performed using factor analysis. The Kaiser-Meyer-Olkin index was 0.86 while the *P-*value for Bartlett’s test was < 0.001. The factor loadings were > 0.3. Cronbach’s alpha for the ASMaQ was 0.82 (13). Both reliability and factor analysis revealed an acceptable reliability and a good construct validity of the scale. The items were scored using a 5-point Likert scale ranging from 1 to 5. The post questionnaire link consisted of Section 3, which measured the post-module knowledge scores using the ASMaQ once again, after the SEM was delivered.

### Statistical Methods

The IBM SPSS Statistics version 27.0 was used for statistical analysis. Pearson’s correlation analysis was done to assess the correlation between pre- and post-module knowledge scores. A paired *t*-test was used to assess the changes in the overall knowledge score and by profession. Knowledge was classified as good or poor based on a previous study (7). Based on expert opinion, a score of 3.5 was considered as the cut-off point for good and poor knowledge, and the score range for the level of knowledge of each domain was classified accordingly (Table S1). The changes in knowledge level from poor to good or vice versa after the SEM was delivered was analysed using the McNemar test. A *P-*value of less than 0.05 was considered as a statistically significant change in knowledge.

### Study Size

Assuming that 5% of the pairs switch from good to poor knowledge and 15% from poor to good knowledge, and after applying continuity corrections, the study would require a minimum sample size of 165 pairs to achieve a power of 80% and a two-sided significance of 5% for detecting a difference of 0.10 between the discordant proportions (14). The total number of participants who completed the pre- and post-module questionnaires during the study period was 171.

## Results

Out of the 567 eligible participants who responded to the pre-module questionnaire, 171 (30.2%) participants completed the post-module questionnaire. The median duration to complete the pre- and post-module questionnaires was approximately 78 days (interquartile range [IQR] = 95.9 days). Most of the participants (56.1%) responded to the post-module questionnaire within 3 months after answering the pre-module questionnaire. About 28% of them responded between 3 months and 6 months, and 15.8% responded after 6 months.

Table 1 shows the demographic characteristics of the participants who completed the pre- and post-module questionnaires. The median (and IQR) for both the age of the participants and the total years of service were 33 (IQR = 7) and 7 (IQR = 7), respectively. Majority of the participants were female (63%), medical officers (71%), working in a hospital (68%), government servants (84%), treating stroke patients in daily practice (85%) and working with specialists (87%).

Pearson’s correlation analysis revealed a significant linear correlation between the pre- and post-module knowledge scores for the domains of GSK (*P* < 0.001), HSM (*P* < 0.001), ASM (*P* < 0.001) and total knowledge scores (*P* < 0.001). The observed correlation coefficients (*r*) ranged from 0.521 to 0.702, indicating a positive and moderate correlation. This showed that the higher the pre-module knowledge scores, the better the post-module knowledge scores would be. Figure S1 depicts a scatter plot of pre- and post-module knowledge scores for each knowledge category.

Table 2 shows the changes in knowledge score during the pre- and post-SEM by the total score and profession. Statistically significant improvements were noted for the ASM knowledge scores (*P* < 0.001) and the total knowledge scores (*P* < 0.001). By profession, significant improvements were noted among the general practitioners for the HSM knowledge scores (*P* = 0.035). According to Table S1, general practitioners’ baseline HSM knowledge scores improved from a poor to a good knowledge range after the SEM was delivered. Significant improvement was also noted among medical officers for the ASM knowledge scores (*P* < 0.001) with a mean difference of a score of 3 and for the total knowledge scores (*P* < 0.001) by a score of 3.8.

### General Stroke Knowledge

**Figure 1:**
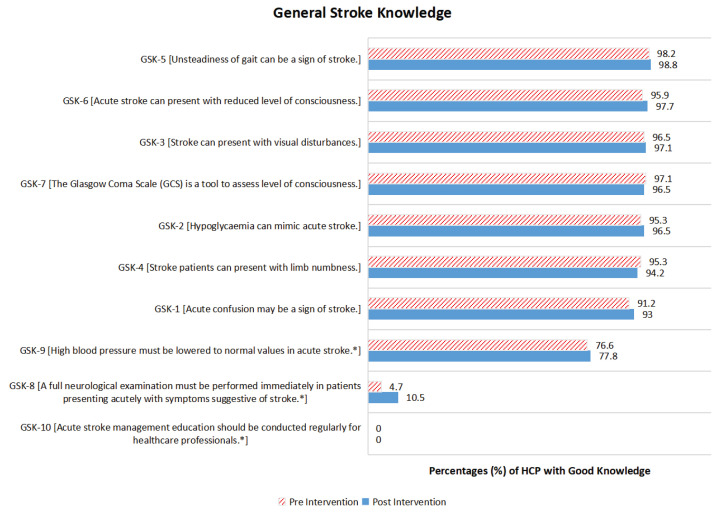
The proportion of participants with good GSK pre- and post-SEM

Items requiring a negative response as the desired response were denoted with asterisks (^*^) for easier result interpretation. Figure 1 shows the proportion of participants with good GSK pre- and post-module SEM. More than 90% of participants already had good knowledge of the signs and symptoms of stroke before the SEM was delivered, and no major changes were noted after the SEM was delivered. Approximately 77% of participants had good baseline knowledge for item GSK-9 [High blood pressure must be lowered to normal values in acute stroke*] and this remained the same after the SEM was delivered. The expected responses for GSK-8 [A full neurological examination must be performed immediately in patients presenting acutely with symptoms suggestive of stroke*] before the SEM was delivered, were very low (4.7%) and increased up to 6% after the SEM was delivered. None of the participants gave the desired answer for item GSK-10 [Acute stroke management education should be conducted regularly for healthcare professionals*].

Table 3 shows the changes in GSK level from poor to good and vice versa during the SEM. For the GSK items, this study showed that more participants had changes in their knowledge level from poor to good compared to good to poor, except for GSK-4, GSK-7 and GSK-10. The proportion of participants with good knowledge was significantly increased post-SEM for item GSK-8 [A full neurological examination must be performed immediately in patients presenting acutely with symptoms suggestive of stroke*] (*P* = 0.021).

### Hyperacute Stroke Management

**Figure 2:**
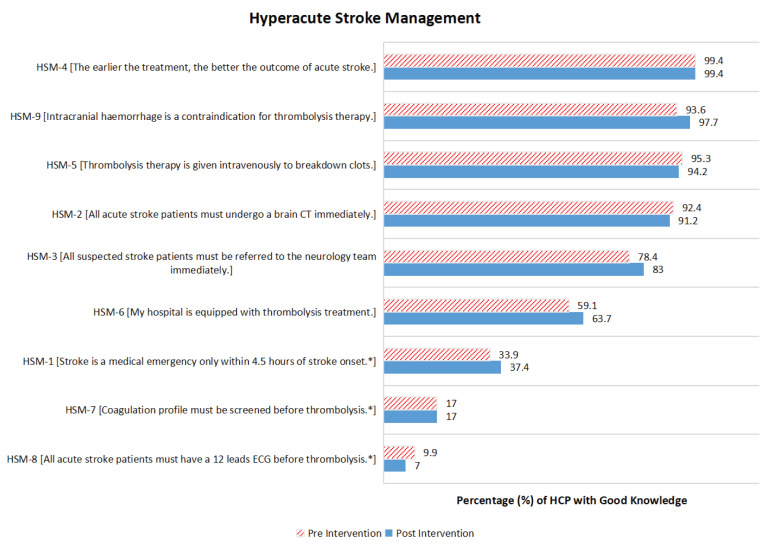
The proportion of participants with good knowledge of HSM during pre- and post-SEM

Figure 2 shows the proportion of participants with good knowledge of HSM during pre- and post-SEM. During pre- and post-SEM, more than 90% of participants correctly responded to items HSM-2 [*All acute stroke patients must undergo a brain CT immediately*], HSM-4 [*The earlier the treatment, the better the outcome of acute stroke*], HSM-5 [*Thrombolysis therapy is given intravenously to breakdown clots*] and HSM-9 [*Intracranial haemorrhage is a contraindication for thrombolysis therapy*]. About 78% of participants had good knowledge of item HSM-3 [*All suspected stroke patients must be referred to the neurology team immediately*]; this increased by 5% post-SEM. About 64% of the participants reported that thrombolysis treatment was available at their workplace after the SEM was delivered. During pre-SEM, only 34% of participants were aware of item HSM-1 [Stroke is a medical emergency only within 4.5 hours of stroke onset*]; this improved slightly to about 3.5% post-SEM. Very few participants had good knowledge for items HSM-8 [All acute stroke patients must have a 12-lead ECG before thrombolysis*] (10%) and HSM-7 [*Coagulation profile must be screened before thrombolysi*s*] (17%) before the SEM was delivered, and no improvements were noted post-SEM.

Table 4 shows the changes in HSM knowledge level from poor to good and vice versa during the SEM. For HSM, majority of the participants changed their knowledge level from a poor to a good knowledge level for items HSM-1, HSM-3, HSM-6 and HSM-9. The knowledge improvement was statistically significant for item HSM-9 [*Intracranial haemorrhage is a contraindication for thrombolysis therapy*] post-SEM (*P* = 0.039).

### Advanced Stroke Management

**Figure 3:**
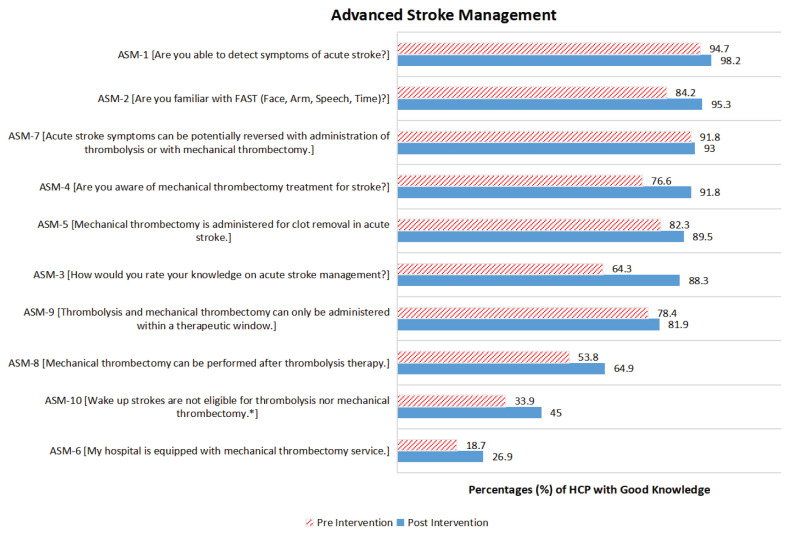
The proportion of participants with good knowledge of ASM during pre- and post-SEM

Figure 3 shows the proportion of participants with good knowledge on ASM during pre- and post-SEM. Most of the participants had good knowledge for the majority of the items under ASM, which ranged from 54% to 95% for pre-SEM and 65% to 98% for post-SEM. Above 10% improvement was noted for items ASM-3 [*How would you rate your knowledge on acute stroke management?*] which was improved by 24%, and ASM-4 [*Are you aware of mechanical thrombectomy treatment for stroke?*] (15%), ASM-8 [*Mechanical thrombectomy can be performed after thrombolysis therapy*] (11%), and ASM-2 [*Are you familiar with FAST (Face, Arm, Speech, Time)?*] (11%). Only 34% of participants gave the desired response for item ASM-10 [Wake up strokes are not eligible for thrombolysis nor mechanical thrombectomy*] during the pre-SEM and it was improved by 11% post-SEM. Approximately 27% of participants were aware that their workplace was equipped with a mechanical thrombectomy service after the SEM.

Table 5 shows the changes in ASM knowledge level from poor to good and vice versa during the SEM. Most of the participants changed their knowledge level from poor to good for all ASM items. Statistically significant improvement was noted for item ASM-2 [*Are you familiar with FAST (Face, Arm, Speech, Time)?]* (*P* < 0.001), ASM-3 [*How would you rate your knowledge on acute stroke management?]* (*P* < 0.001), ASM-4 [*Are you aware of mechanical thrombectomy treatment for stroke?]* (*P* < 0.001), ASM-5 [*Mechanical thrombectomy is administered for clot removal in acute stroke*] (*P* = 0.013), ASM-6 [*My hospital is equipped with mechanical thrombectomy service*] (*P* = 0.013), ASM-8 [*Mechanical thrombectomy can be performed after thrombolysis therapy*] (*P* = 0.008) and ASM-10 [Wake up strokes are not eligible for thrombolysis nor mechanical thrombectomy*] (*P* = 0.016).

### Overall Knowledge

**Figure 4:**
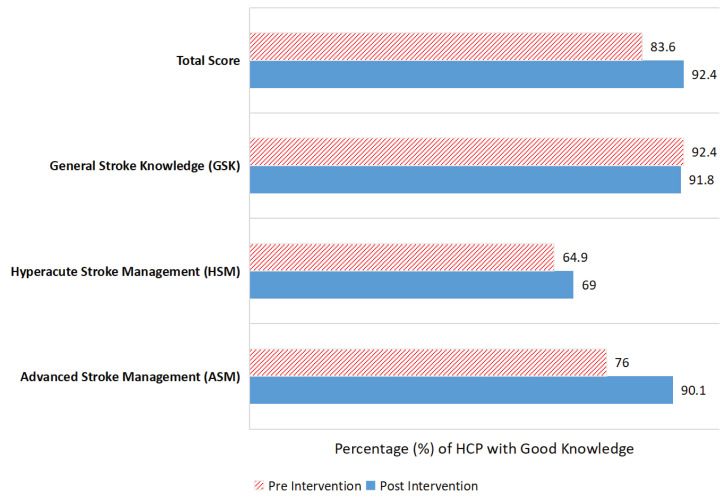
The changes in overall total knowledge during the SEM

Figure 4 and Table 6 show the changes in overall total knowledge during the SEM. The proportion of participants who improved their knowledge level from poor to good was higher (12.3%) compared to those who deteriorated from good to poor (3.5%). The net improvement of 8.8% was statistically significant (*P* = 0.006). For the subgroup knowledge score, a statistically significant change was noted for ASM knowledge (*P* < 0.001). About 17.5% of participants improved their knowledge from poor to good, while only 3.5% deteriorated, resulting in a net improvement of 14%. No significant changes were noted for the subgroups GSK (-0.6%) (*P* = 1.000) and HSM (4.1%) (*P* = 0.435).

## Discussion

This is the first study to assess the effectiveness of SEM among doctors in Malaysia. The response rate (30%) was lower than the average online survey response rate of 44% reported in a meta-analysis of published education-related research (15). However, the distribution of characteristics among participants who responded to the post-module questionnaire was similar to that of those who answered the pre-module questionnaire in the earlier study (7).

Both numerical and categorical knowledge score analyses showed a significant improvement in ASM knowledge and total knowledge scores, particularly among medical officers. Generally, for each category of knowledge, the baseline knowledge scores for all the professions were within a good knowledge level range, except for the general practitioner’s HSM knowledge score. Even though there is no overall improvement in HSM knowledge scores, the general practitioners’ knowledge improved following the SEM. This showed that SEM was still beneficial in improving doctors’ knowledge to a good level, although clinically there was not much improvement in the mean score differences.

This study showed that SEM significantly improved doctors’ knowledge of ASM, particularly mechanical thrombectomy. More doctors were aware of mechanical thrombectomy, its indications and the availability of these services at their hospital. After the SEM, more doctors were familiar with the F.A.S.T. acronym to recognise stroke symptoms. Despite a significant increase in doctors’ knowledge of wake-up stroke eligibility for reperfusion therapy following the SEM, 44% of doctors’ knowledge remained poor; thus, this area of knowledge still requires improvement. Wake-up stroke is still eligible for mechanical thrombectomy, as the treatment window can be extended via CT or MR perfusion (penumbra-core mismatch) or MRI (clinical-imaging mismatch) up to 24 h from the time the patient was last known to be well (6). They are also eligible for thrombolysis in the presence of a DWI-FLAIR mismatch via MRI if the onset is uncertain (6). Regardless, after the SEM, doctors’ perceptions of their knowledge of acute stroke management improved significantly from 64% to 88%. In a previous study, 95.6% of emergency medical services (EMS) professionals in the United Kingdom reported that online courses improved their knowledge on acute stroke management, which was higher than in this study (16). This was probably due to the EMS professionals’ scope of the training, which might be different or more specific to their field compared to this module. The SEM was more general and open to all doctors, regardless of their field of expertise. Furthermore, only 18% of the participants in this study were EMS professionals.

There was no improvement noted for GSK because the baseline knowledge of the participants prior to the SEM was excellent, particularly regarding the signs and symptoms of stroke, indicating a high level of awareness of stroke symptoms among Malaysian doctors. Similarly, another study among EMS professionals found no significant improvement in knowledge for the signs and symptoms of stroke questions after an online training program because their baseline knowledge was already excellent (99.6%) (11). Nonetheless, special attention should be given to the knowledge regarding the initial assessment and management of acute stroke. Although there was a statistically significant improvement for item GSK-8 [A full neurological examination must be performed immediately in patients presenting acutely with symptoms suggestive of stroke*], 88% of the doctors’ knowledge remained poor. Despite the availability of the National Institutes of Health Stroke Scale (NIHSS), which enables a quick assessment of acute stroke patients, most doctors continued to believe that a full neurological examination is required for acute stroke patients. Timing is crucial in the treatment of an acute stroke patient. Doctors should be made aware of the importance of a prompt assessment of stroke patients to prevent treatment delays that can reduce the chance of a successful treatment. Our study also found that no significant changes in responses were noted for GSK-9 [High blood pressure must be lowered to normal values in acute stroke*]. Even though the baseline knowledge was satisfactory (77%), it still can be improved. Uncontrolled hypertension is an exclusion criterion for thrombolysis only when the values exceed a systolic blood pressure of 185 mmHg and/or a diastolic blood pressure of 110 mmHg (17). Hence, maintaining a blood pressure below these values is sufficient; reducing it to normal values is unnecessary and will merely delay the treatment. Future training should emphasise the pre-thrombolysis patient assessment and management, such as NIHSS assessment and blood pressure control in acute stroke. Apart from that, none of the doctors provided the desired response for the item GSK-10 [Acute stroke management education should be conducted regularly for healthcare professionals*]. Initially, participants were expected to disagree with the idea that healthcare professionals should regularly receive education on acute stroke management if they have already acquired adequate knowledge. However, this was an ambiguous question. The doctors most likely agreed with this item because, generally, regular education can improve doctors’ knowledge.

Our study also showed that there was no significant improvement in overall HSM knowledge, after the SEM. Even though the participants’ knowledge of most of the HSM items was already good at baseline, some of them remained with very poor knowledge level after the SEM. Especially for items HSM-7 [*Coagulation profile must be screened before thrombolysi*s*] and HSM-8 [All acute stroke patients must have a 12-lead ECG before thrombolysis*], even after the SEM, most doctors still considered that the coagulation profile (76%) and 12-lead electrocardiogram (87%) were prerequisite investigations for thrombolysis. The initiation of thrombolysis should not be delayed while awaiting these tests’ results. Coagulation profile test results are only required if the patient has a high tendency to bleed or is receiving anticoagulant therapy. For item HSM-1 [Stroke is a medical emergency only within 4.5 hours of stroke onset*], approximately 51% of them remained unaware of the indications for thrombolysis in the extended window beyond 4.5 h. The current SEM covers HSM in one module (Module 5: Hyperacute management and decision making). Future training should include more HSM-related modules, particularly covering topics such as the prerequisite investigation and indication of thrombolysis in the extended window period.

The SEM increased overall knowledge of acute stroke management from 84% to 92% across all subgroups of stroke knowledge. This was mainly due to the high baseline GSK score and the significant improvement in ASM knowledge scores following the SEM.

### Advantages and Disadvantages of the Stroke e-Learning Module

This stroke module was pre-recorded and was made available online. The advantages of having pre-recorded videos were that it might have better content clarity and can become a reusable learning tool. Since it was accessible online, it could be viewed anytime and anywhere at the learner’s own pace. This was especially beneficial for HCPs who have a heavy workload and usually cannot allocate specific time for training. The e-learning module also has no limits on the number of learners that can be reached. In addition, online training was extremely beneficial, particularly during a pandemic such as COVID-19, where social distancing was needed. Nonetheless, this module did have some disadvantages. Since it was pre-recorded, there was no interaction between the speakers and learners. The learners were unable to ask questions or clarify their uncertainties. This online module may also be challenging to access in remote areas with limited internet access. Stroke care is an evolving area where new evidence may become available, and the content of the pre-recorded lectures may become irrelevant in the future. Therefore, healthcare professionals should always seek the latest management recommendations for stroke.

### Limitations and Recommendations

There are several limitations in this study. The first limitation is that the pre- and post-module test study design lacked a control group, which may affect the internal validity of this study. Due to the lack of random assignment, this study was susceptible to selection bias. Furthermore, participants were subject to maturation, which meant that they could learn more knowledge over time, either naturally or through other modes of learning that occurred concurrently. Regression to the mean could have also occurred in this study design. It is a statistical phenomenon that can occur when the pre-module test score is extremely high, and the post-test score eventually regresses to the mean. This was true for several items in this study, including GSK-4, GSK-7, HSM-2 and HSM-5, which had very high baseline scores but slightly lower post-module test scores. Therefore, future research should consider adding a control group to reduce these effects and biases. Secondly, the pre- and post-module knowledge levels were measured at different times because there was no specific deadline for completing the module and the post-module questionnaire. The knowledge level of those who responded to the post-module questionnaire immediately after the module may differ from that of those who responded later, as their knowledge sustainability may have diminished. Future training modules therefore should track the time spent by the participant to assure training completion, and further research should be done to assess their knowledge retention. Thirdly, some of the items in the questionnaire did not directly measure knowledge of AIS management. For instance, items HSM-6 and ASM-6 asked about the availability of thrombolysis and thrombectomy services at their hospitals. These items did not directly reflect their understanding of stroke management. Those who work in the healthcare facility without the services would have received a lower score, even if they were knowledgeable in stroke management. In addition, this study did not request for the participants’ institutional information, which precluded the ability to verifying their responses to these items. Fourthly, this study also did not request the participants’ evaluation of the online module itself, which would have helped to assess the module’s feasibility and be beneficial in refining the module for future training. Lastly, the knowledge level reported in this study might not necessarily reflect the actual practice of the HCPs. Future research should evaluate the changes in practice among HCPs who participated in this training module.

## Conclusion

The SEM has been shown to increase Malaysian doctors’ knowledge on acute stroke management, particularly in ASM. However, greater effort should be made to improve the GSK and HSM, especially in areas related to the indication for stroke thrombolysis. The SEM should be continuously improved based on the knowledge gaps identified in this study and subsequent research.

## Figures and Tables

**Table 1:** Demographic characteristics of participants (*n* = 171)

Variables	*n* (%)	Median (IQR)
Age (years old)		33 (7)
Service (years)		7 (7)
< 5	48 (28.1)	
5–10	75 (43.9)	
> 10	48 (28.1)	
Gender		
Male	64 (37.4)	
Female	107 (62.6)	
Professions		
Specialist	36 (21.1)	
Medical officer	121 (70.8)	
General practitioner	14 (8.2)	
Working field/Department		
Emergency	30 (17.5)	
Medical	65 (38.0)	
Primary care	55 (32.2)	
Others	21 (12.3)	
Work setting		
Primary care	55 (32.2)	
Hospital	116 (67.8)	
Type of primary care		
Government health clinic	39 (70.9)	
Private clinic	16 (29.1)	
Type of hospital		
Private hospital	11 (9.5)	
Public hospital with specialist	51 (44.0)	
Public hospital without specialist	43 (37.1)	
University hospital	11 (9.5)	
Work sector		
Government	144 (84.2)	
Private	27 (15.8)	
Seeing stroke patients in daily practice		
Yes	145 (84.8)	
No	26 (15.2)	
Workplace with specialists		
Yes	148 (86.5)	
No	23 (13.5)	

Note: IQR = interquartile range

**Table 2:** The changes in knowledge score during the pre- and post-SEM as an overall score and by profession (paired *t*-test)

Knowledge domains	Professions	Pre-intervention		Post-intervention		Mean of score difference (95% CI)		*t* statistic (df)		*P-*value
		mean	(SD)	mean	(SD)					
General stroke knowledge (GSK)	Overall	39.7	(3.16)	39.9	(3.63)	0.2	(−0.3, 0.7)	0.6	(170)	0.523
	Specialist	40.6	(2.58)	40.1	(5.14)	−0.4	(−2.0,1.2)	−0.5	(35)	0.596
	Medical officer (MO)	39.6	(3.26)	40.0	(3.04)	0.4	(−0.1,0.9)	1.6	(120)	0.12
	General practitioner (GP)	38.6	(3.34)	38.1	(3.48)	−0.5	(−2.1,1.1)	−0.7	(13)	0.511
Hyperacute stroke management (HSM)	Overall	33.1	(3.89)	33.3	(3.85)	0.2	(−0.4, 0.7)	0.7	(170)	0.504
	Specialist	35.3	(4.56)	34.9	(4.24)	−0.4	(−1.6,0.9)	−0.6	(35)	0.569
	MO	32.8	(3.57)	33.0	(3.77)	0.2	(−0.5,0.8)	0.5	(120)	0.589
	GP	30.6	(2.06)	32.3	(2.23)	1.6	(0.1,3.2)	2.3	(13)	0.035
Advanced stroke management (ASM)	Overall	38.3	(4.98)	40.8	(4.61)	2.5	(1.8, 3.2)	7.2	(170)	< 0.001
	Specialist	41.4	(4.42)	42.6	(3.49)	1.1	(0.0,2.3)	2.0	(35)	0.056
	MO	37.7	(4.86)	40.7	(4.68)	3.0	(2.1,3.8)	7.1	(120)	< 0.001
	GP	35.3	(3.71)	37.1	(4.29)	1.8	(−1.6,5.1)	1.2	(13)	0.269
Total knowledge score	Overall	111.2	(9.37)	114.0	(8.81)	2.8	(1.8, 3.9)	5.3	(170)	< 0.001
	Specialist	117.25	(9.66)	117.61	(8.78)	0.4	(−2.2,2.9)	0.3	(35)	0.773
	MO	110.1	(8.79)	113.68	(8.57)	3.6	(2.3,4.8)	5.7	(120)	< 0.001
	GP	104.57	(5.00)	107.5	(6.70)	2.9	(−0.4,6.3)	1.9	(13)	0.08

**Table 3:** The changes in knowledge level during pre- and post-SEM for GSK (McNemar test)

	Changes of knowledge level, *n* (%)				*X*^2^ (df)	*P-*value

Pre-intervention	Good	Good	Poor	Poor		

Post-intervention	Good	Poor	Good	Poor		
1. Acute confusion may be a sign of stroke	146 (85.4)	10 (5.8)	13 (7.6)	2 (1.2)	0.39 (1)	0.678
2. Hypoglycaemia can mimic acute stroke	159 (93.0)	4 (2.3)	6 (3.5)	2 (1.2)	0.40 (1)	0.754
3. Stroke can present with visual disturbances	161 (94.2)	4 (2.3)	5 (2.9)	1 (0.6)	0.11 (1)	1.000
4. Stroke patients can present with limb numbness	156 (91.2)	7 (4.1)	5 (2.9)	3 (1.8)	0.33 (1)	0.774
5. Unsteadiness of gait can be a sign of stroke	167 (97.7)	1 (0.6)	2 (1.2)	1 (0.6)	0.33 (1)	1.000
6. Acute stroke can present with reduced level of consciousness	162 (94.7)	2 (1.2)	5 (2.9)	2 (1.2)	1.29 (1)	0.453
7. The Glasgow Coma Scale (GCS) is a tool to assess level of consciousness	161 (94.2)	5 (2.9)	4 (2.3)	6 (3.5)	0.11 (1)	1.000
8. A full neurological examination must be performed immediately in patients presenting acutely with symptoms suggestive of stroke*	5 (2.9)	3 (1.8)	13 (7.6)	150 (87.7)	6.25 (1)	0.021
9. High blood pressure must be lowered to normal values in acute stroke*	114 (66.7)	17 (9.9)	19 (11.1)	21 (12.3)	0.11 (1)	0.868
10. Acute stroke management education should be conducted regularly for healthcare professionals*	0	0	0	171 (100)	–	–

Note: The italicised and asterisked (*) items denoted the items that required a negative response as desired responses

**Table 4:** The changes in knowledge level during pre- and post-SEM for HSM (McNemar test)

	Changes of knowledge level, *n* (%)				*X^2^* (df)	*P-*value

Pre-intervention	Good	Good	Poor	Poor		

Post-intervention	Good	Poor	Good	Poor		
1. Stroke is a medical emergency only within 4.5 h of stroke onset*	38 (22.2)	20 (11.7)	26 (15.2)	87 (50.9)	0.78 (1)	0.461
2. All acute stroke patients must undergo a brain CT immediately	148 (86.5)	10 (5.8)	8 (4.7)	5 (2.9)	0.22 (1)	0.815
3. All suspected stroke patients must be referred to the neurology team immediately	123 (71.9)	11 (6.4)	19 (11.1)	18 (10.5)	2.13 (1)	0.200
4. The earlier the treatment, the better the outcome of acute stroke	169 (98.8)	1 (0.6)	1 (0.6)	0	0.00 (1)	1.000
5. Thrombolysis therapy is given intravenously to breakdown clots	154 (90.1)	9 (5.3)	7 (4.1)	1 (0.6)	0.25 (1)	0.804
6. My hospital is equipped with thrombolysis treatment	88 (51.5)	13 (7.6)	21 (12.3)	49 (28.7)	1.88 (1)	0.229
7. Coagulation profile must be screened before thrombolysis*	17 (9.9)	12 (7.0)	12 (7.0)	130 (76.0)	0.00 (1)	1.000
8. All acute stroke patients must have a 12-leads ECG before thrombolysis*	6 (3.5)	11 (6.4)	6 (3.5)	148 (86.5)	1.47 (1)	0.332
9. Intracranial haemorrhage is a contraindication for thrombolysis therapy	159 (93.0)	1 (0.6)	8 (4.7)	3 (1.8)	5.44 (1)	0.039

Note: The italicised and asterisked (*) items denoted the items that required a negative response as desired responses

**Table 5:** The changes in knowledge level during pre and post-SEM for ASM (McNemar test)

	Changes of knowledge level, *n* (%)				*X^2^* (df)	*P-*value

Pre-intervention	Good	Good	Poor	Poor		

Post-intervention	Good	Poor	Good	Poor		
1. Are you able to detect symptoms of acute stroke?	160 (93.6)	2 (1.2)	8 (4.7)	1 (0.6)	3.60 (1)	0.109
2. Are you familiar with FAST (Face, Arm, Speech, Time)?	144 (84.2)	0	19 (11.1)	8 (4.7)	19.00 (1)	< 0.001
3. How would you rate your knowledge on acute stroke management?	106 (62.0)	4 (2.3)	45 (26.3)	16 (9.4)	34.31 (1)	< 0.001
4. Are you aware of mechanical thrombectomy treatment for stroke?	126 (73.7)	5 (2.9)	31 (18.1)	9 (5.3)	18.78 (1)	< 0.001
5. Mechanical thrombectomy is administered for clot removal in acute stroke	132 (77.2)	7 (4.1)	21 (12.3)	11 (6.4)	7.00 (1)	0.013
6. My hospital is equipped with mechanical thrombectomy service	25 (14.6)	7 (4.1)	21 (12.3)	118 (69.0)	7.00 (1)	0.013
7. Acute stroke symptoms can be potentially reversed with administration of thrombolysis or with mechanical thrombectomy	146 (85.4)	11 (6.4)	13 (7.6)	1 (0.6)	0.17 (1)	0.839
8. Mechanical thrombectomy can be performed after thrombolysis therapy	78 (45.6)	14 (8.2)	33 (19.3)	46 (26.9)	7.68 (1)	0.008
9. Thrombolysis and mechanical thrombectomy can only be administered within a therapeutic window	122 (71.3)	12 (7.0)	18 (10.5)	19 (11.1)	1.20 (1)	0.362
10. Wake up strokes are not eligible for thrombolysis nor mechanical thrombectomy*	39 (22.8)	19 (11.1)	38 (22.2)	75 (43.9)	6.33 (1)	0.016

Note: The italicised and asterisked (*) items denoted the items that required a negative response as desired responses

**Table 6:** The changes in overall knowledge level during pre- and post-SEM (McNemar test)

	Changes of knowledge level, *n* (%)				*X^2^* (df)	*P-*value

Pre-intervention	Good	Good	Poor	Poor		

Post-intervention	Good	Poor	Good	Poor		
General stroke knowledge (GSK)	150 (87.7)	8 (4.7)	7 (4.1)	6 (3.5)	0.07 (1)	1.000
Hyperacute stroke management (HSM)	85 (49.7)	26 (15.2)	33 (19.3)	27 (15.8)	0.83 (1)	0.435
Advanced stroke management (ASM)	124 (72.5)	6 (3.5)	30 (17.5)	11 (6.4)	16.00 (1)	< 0.001
Total knowledge score	137 (80.1)	6 (3.5)	21 (12.3)	7 (4.1)	8.33 (1)	0.006

## Appendix

### Stroke e-Learning Module

#### Module 1: Stroke Pathophysiology and Clinical Features

- Speaker: Dr. Abdul Hanif Khan Yusof Khan (Internal Medicine Physician and Neurology Fellow, Hospital Pengajar UPM)
- Summary content: This module emphasizes the pathophysiology of stroke and the clinical features based on the pathophysiology.
- Objectives of the module:
  1. Participants will be able to define stroke and the timing of a stroke.
  2. Participants will understand the pathophysiology of stroke.
  3. Participants will determine the clinical features of stroke and classify them into OXFORD and TOAST classification.

#### Module 2: Stroke Mechanism in Transient Ischaemic Attack (TIA) and Mild Stroke

- Speaker: Dr. Wan Asyraf Wan Zaidi (Consultant Physician and Neurologist, Hospital Chancellor Tengku Mukhriz)
- Summary content: This module will emphasize the pathophysiology and management of TIA and mild stroke.
- Objectives of the module:
  1. Participants will be able to identify TIAs and a mild stroke.
  2. Participants will understand the urgency to prevent major stroke in TIAs and mild stroke and to manage the associated risk factors.

#### Module 3: Stroke Mimics

- Speaker: Associate Prof Dr. Wan Nur Nafisah Wan Yahya (Consultant Physician and Neurologist, Hospital Chancellor Tengku Mukhriz)
- Summary content: This module emphasize on stroke mimics, stroke chameleons, and a step-by-step approach to distinguish stroke from its mimics
- Objectives of the module:
  1. Participants will be able to recognize a stroke mimic
  2. Participants will be able not to miss stroke chameleons
  3. Participants will be able to assess and investigate patients to rule out stroke mimics

#### Module 4: Pre-Hospital Care

- Speaker: Dr. Sarah Abdul Karim (Consultant Emergency Physician-Hospital Sungai Buloh)
- Summary content: This module emphasizes the role of pre-hospital care (mainly stroke recognition among paramedics) and methods to optimize emergency care in stroke, particularly time management.
- Objectives of the module:
  1. Participants will be able to understand stroke patients’ journey from pre-hospital care.
  2. Participants will be able to identify the stroke scale used in pre-hospital to identify stroke and stratify accordingly.
  3. Participants will understand the emergency physician’s role in the emergency setting as the first liner to manage and stabilize stroke patients.

#### Module 5: Hyperacute Management and Decision Making

- Speaker: Dr Law Wan Chung (Consultant Physicians and Neurology, Hospital Umum Sarawak)
- Summary of content: This module will emphasize the steps taken to determine stroke patients’ management in the hyperacute phase.
- Objectives of the module:
  1. Participants will be able to assess patients in the hyperacute phase for the suitability of intravenous thrombolysis by the following criteria i.e.
    b. Making the diagnosis
    c. Exclude bleed
    d. Assess severity
    e. Identify contraindications
  2. Participants will be able to determine the ASPECT and NIHSS scoring.

#### Module 6: Imaging in Stroke

- Speaker: Associate Professor Dr. Hiwati Hashim (Consultant Neuroradiologist, Hospital UiTM)
- Summary of content: This module will emphasize the basic CT scan reading on identifying crucial abnormalities in particular intra-cranial bleed and identifying signs of early stroke in CT scan.
- Objectives of the module:
  1. Participants will be able to identify normal CT scan brain.
  2. Participants will be able to determine bleed on the CT scan brain.
  3. Participants will able to determine the early signs of a stroke on CT brain and determine the ASPECT score.

#### Module 7: Post-Acute Care in Stroke

- Speaker: Dr. Khairul Azmi Ibrahim (Consultant Physician and Neurologist, Hospital Sultanah Zanariah Terengganu)
- Summary of content: This module will emphasize post-acute care in stroke patients.
- Objectives of the module:
  1. Participants will be able to determine the general management post-stroke.
  2. Participants will be able to understand the importance of stroke unit and organized stroke care.
  1. Participants will be able to determine the monitoring required in particular post intravenous thrombolysis, and treat the associated complications.

#### Module 8: Secondary Prevention of Stroke

- Speaker: Dr. Tan Wee Yong (Consultant Physicians and Neurologist, Thomson Hospital, Kuala Lumpur)
- Summary content: This module emphasizes post-stroke management mainly on antiplatelet agents, hypertensive, hyperlipidemia, and blood sugar control post-stroke.
- Objectives of the module:
  1. Participants will be able to determine the role of single and double antiplatelet agents.
  2. Participants will be able to determine the optimum blood pressure, lipid level, and blood glucose control post-stroke.
  3. Participants will understand the role of lifestyle modification, in particular, smoking cessation.
  4. Participants will be able to understand the role of identifying risk factors e.g., atrial fibrillation or carotid artery stenosis.

#### Module 9: Primary Prevention of Stroke

- Speaker: Associate Professor Dr. Ching Siew Mooi (Consultant Family Medicine, Hospital Pengajar UPM)
- Summary content: This module will emphasize the primary prevention of stroke.
- Objectives of the module:
  1. Participants will be able to understand the current guideline in stroke prevention.
  2. Participants will be able to assess patients and manage the risk factors associated with stroke, mainly hypertension, hyperlipidemia, diabetic control, and lifestyle (e.g., smoking).

#### Module 10: NIHSS Training

- Speaker: Dr. Irene Looi (Consultant Physician and Neurologist Hospital Seberang Jaya, Pulau Pinang)
- Summary Content: This module will talk on the NIHSS (National Institue Health Stroke Scale) as a surrogate score to determine the stroke severity and may aid during the hyperacute management decision.
- Objectives of the module:
  1. Participants will be familiar with the NIHSS (National Institute of Health Stroke Scale) and its component.
  2. Participants will be able to score the patient NIHSS score after examination of stroke patients.

Figure S1 Scatter plots of pre- and post-module knowledge scores for each knowledge category

**Table S1:** Classification of the level of knowledge

Levels of knowledge	Likert scale responses#	Range of scores calculation	Overall	GSK	HSM	ASM
	(Cut-off point = 3.5)		(29 items)	(10 items)	(9 items)	(10 items)
Good	4 & 5	≥ (Total number of items × 3.5)	101.5–145	35–50	31.5–45	35–50
Poor	1, 2 & 3	< (Total number of items × 3.5)	29–101.5	10–35	9–31.5	10–35

Note:

# After reverse coding done for negative answers as desired response
