# Supplementary figures and images for: Effectiveness of the Stroke e-Learning Module on Malaysian Doctors’ Knowledge of Acute Ischaemic Stroke Management
Source: Malays J Med Sci. 2024 Aug 27;31(4):195–212. doi: 10.21315/mjms2024.31.4.16 (PMC11377012; doi:10.21315/mjms2024.31.4.16)

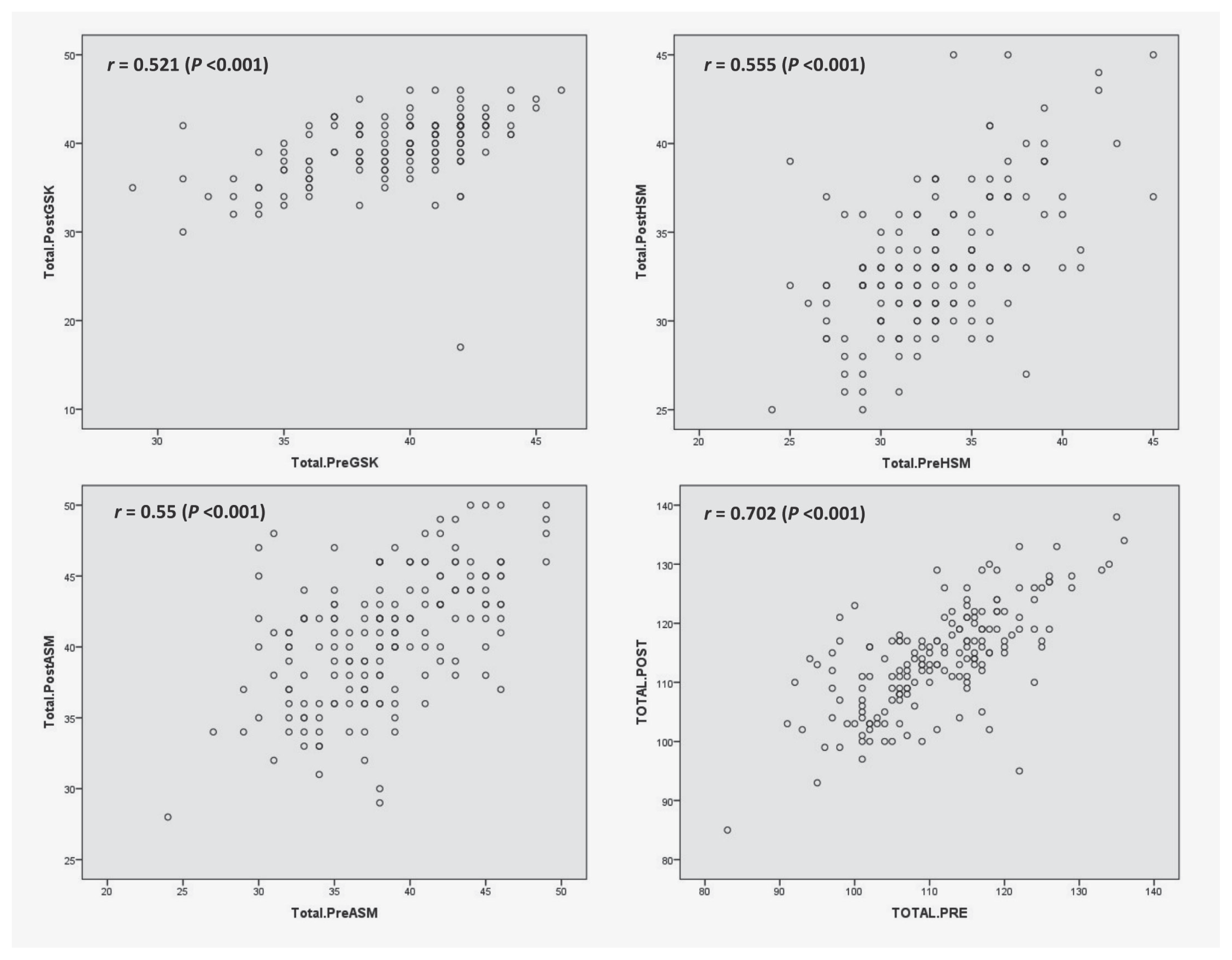

Supplement: Figure S1 — Scatter plots of pre- and post-module knowledge scores for each knowledge category [file 16mjms3104_oas1.tif]
